# Novel Microdeletion in the X Chromosome Leads to Kallmann Syndrome, Ichthyosis, Obesity, and Strabismus

**DOI:** 10.3389/fgene.2020.00596

**Published:** 2020-06-24

**Authors:** Wanlu Ma, Jiangfeng Mao, Xi Wang, Lian Duan, Yuwen Song, Xiaolan Lian, Junjie Zheng, Zhaoxiang Liu, Min Nie, Xueyan Wu

**Affiliations:** ^1^Department of Endocrinology, Peking Union Medical College Hospital, Chinese Academy of Medical Sciences, Beijing, China; ^2^Department of Endocrinology, The Second Hospital of Shandong University, Jinan, China; ^3^Department of Endocrinology, Beijing Tsinghua Changgung Hospital, School of Clinical Medicine, Tsinghua University, Beijing, China

**Keywords:** Kallmann syndrome, X-linked ichthyosis, X chromosome microdeletion, obesity, strabismus

## Abstract

**Background:**

A large deletion in Xp22.3 can result in contiguous gene syndromes, including X-linked ichthyosis (XLI) and Kallmann syndrome (KS), presenting with short stature, chondrodysplasia punctata, intellectual disability, and strabismus. XLI and KS are caused by the deletion of *STS* and *ANOS1*, respectively.

**Method:**

Two KS patients with XLI were screened to identify possible pathogenic mutations using whole exome sequencing. The clinical characteristics, molecular genetics, treatment outcomes, and genotype–phenotype association for each patient were analyzed.

**Results:**

We identified a novel 3,923 kb deletion within the Xp22.31 region (chrX: 5810838–9733877) containing *STS*, *ANOS1*, *GPR143*, *NLGN4X*, *VCX-A*, *PUDP*, and *PNPLA4* in patient 1, who presented with KS, XLI, obesity, hyperlipidemia, and strabismus. We identified a novel 5,807 kb deletion within the Xp22.31-p22.33 regions (chrX: 2700083–8507807) containing *STS*, *ANOS1*, and other 24 genes in patient 2, who presented with KS, XLI, obesity, and strabismus. No developmental delay, abnormal speech development, or autistic behavior were noticed in either patient.

**Conclusion:**

We identified two novel microdeletions in the X chromosome leading to KS and XLI. These findings contribute to the understanding of the molecular mechanisms that drive contiguous gene syndromes. Our research confirmed that the Kallmann-Ichthyosis phenotype is caused by microdeletions at the chromosome level.

## Introduction

**FIGURE 1:**
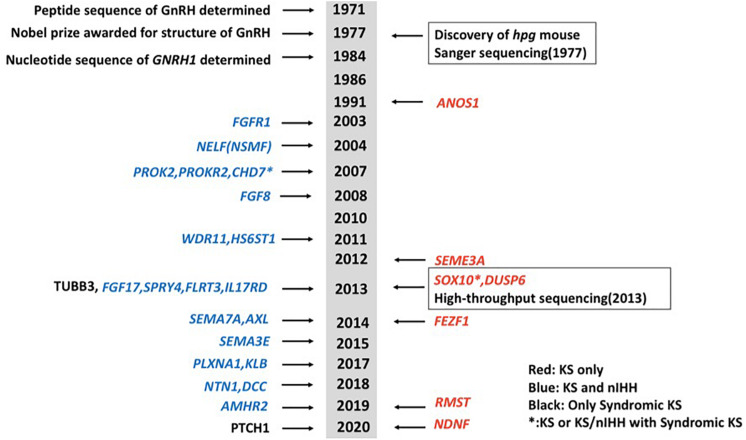
A brief chronology of genes discovered for Kallmann syndrome (KS). Genes in red show genes that only cause KS. Genes in blue show genes that can cause both KS and normosmic idiopathic hypogonadotropic hypogonadism (nIHH). Two genes in black mean they cause syndromic diseases including KS. Genes with asterisk show they can cause not only KS or KS/nIHH but also syndromic diseases including KS.

Idiopathic hypogonadotropic hypogonadism (IHH) is a genetically heterogeneous disorder, which can be classified into normosmic idiopathic hypogonadotropic hypogonadism (nIHH) and Kallmann syndrome (KS, MIM 147950, 244200, 308700, 610628, 612370, and 612702) (Maya-Nuñez et al., 1999; Trarbach et al., 2005; Köhn and Schuppe, 2014; Boehm et al., 2015; Bonomi et al., 2018; Stamou and Georgopoulos, 2018). KS, which is caused by a gonadotrophin-releasing hormone (GnRH) deficiency and hypoplasia of the olfactory bulbs (Maya-Nuñez et al., 1999; Trarbach et al., 2005; Mitchell et al., 2011; Köhn and Schuppe, 2014; Stamou and Georgopoulos, 2018), is characterized by hypogonadotropic hypogonadism, anosmia or hyposmia, and non-reproductive phenotypes including mirror movement, unilateral renal agenesis, eye movement disorders, hearing loss, and cleft lip/palate. KS has a prevalence of around 1:8,000 in men and 1:40,000 in women (de Castro et al., 2017). Mutations in genes that disrupt the development and migration of GnRH neurons may cause KS (Stamou and Georgopoulos, 2018). Current known mutations in various genes are accountable for ∼40% of cases of IHH (Bonomi et al., 2018). While mutations in some genes primarily cause KS and some cause nIHH only, mutations in some other genes have been linked to both KS and nIHH. Gene mutations that are only present in KS probands include *ANOS1* (alias *KAL1*), *SOX10*, *SEMA3A*, *FEZF1*, *DUSP6*, *RMST*, and *NDNF* (Miraoui et al., 2013; Messina et al., 2020; Stamou et al., 2020). Genes that can cause both KS and nIHH include *FGFR1*, *NSMF*, *PROK2*, *PROKR2*, *CHD7*, *FGF8*, *WDR11*, *HS6ST1*, *FGF17*, *IL17RD*, *SPRY4*, *FLRT3*, *SEMA7A*, *AXL*, *SEMA3E*, *PLXNA1*, *KLB*, *NTN1*, *DCC*, and *AMHR2* (Abreu et al., 2008; Canto et al., 2009; Miraoui et al., 2013; Stamou et al., 2016; Marcos et al., 2017; Xu et al., 2017; Bouilly et al., 2018; Maione et al., 2018; Malone et al., 2019; Young et al., 2019). Mutations in TUBB3 and PTCH1 may also cause syndromic diseases including KS (Mitchell et al., 2011; Barraud et al., 2020). A brief chronology of genes linked to KS was summarized (Figure 1). KS can be inherited in either an X-linked recessive, autosomal dominant, or autosomal recessive pattern, often associated with chromosomal abnormalities including lp21.1, 2q32.2, 8q21.13, 14q21.2, and Xp22.31 (Hamada et al., 2013). *ANOS1*, located at the Xp22.3 locus, encodes the protein anosmin-1 and was the first gene found to cause X-linked KS.

Ichthyoses comprises a heterogeneous group of genetic disorders characterized by cornification and scaling of the skin, with either an autosomal or X-linked inheritance pattern. X-linked ichthyosis (XLI, OMIM:308100), which typically results in the early onset of dark, dry, and irregular scales affecting the limb and trunk, is caused by the deletion of the *STS* gene, encoding steroid sulfatase and located near *ANOS1* at Xp22.3 (Takeichi and Akiyama, 2016).

Chromosomal rearrangements, especially large terminal or interstitial deletions of Xp22.3, have been described in patients with various disease associations known as contiguous gene syndromes. Abnormalities in Xp22.3 can result in short stature, chondrodysplasia punctata, intellectual disability, strabismus, XLI, and KS. The Xp 22.31 region contains several genes, including *STS*, *ANOS1*, *NLGN4X*, *HDHD1* (*PUDP*), *PNPLA4*, and the *VCX* cluster. Submicroscopic deletions of *STS* and *ANOS1* may lead to KS and XLI (Nagai et al., 2017). However, the genomic bases of other microdeletions within the Xp22.31 region remain unclear. We, therefore, sought to detect and confirm the pathogenic mutations for two patients presenting with both KS and ichthyosis.

## Materials and Methods

### Subjects

Two KS patients with XLI were recruited from the Department of Endocrinology, Peking Union Medical College Hospital (PUMCH) from January 2002 to July 2019 and screened. This study was approved by the Ethics Committee of PUMCH. Signed informed consent was obtained from both patients.

### Clinical Evaluation

In both cases, a thorough medical history was collected. A thorough physical examination was carried out, including both patients’ height, weight, skin condition, and external genital examination. Laboratory assessments included analysis of sex hormones, anterior pituitary hormones, and a triptorelin-stimulating test. Radiographic imaging assessments included an olfactory bulb and tract nerve MRI, a pituitary MRI, and a urinary ultrasound. Luteinizing hormone (LH), follicle-stimulating hormone (FSH), and testosterone levels were measured via a chemiluminescent method using a commercial kit (ACS 180 Automatic Chemiluminescence System; Bayer, Germany). Testicular size was measured using a Prader orchidometer.

### Genetic Analyses

Genomic DNA was extracted from peripheral white blood cells using a DNA Extraction Kit (QIAamp DNA; Qiagen, Germany). Pathogenic mutations were identified using whole exome sequencing (MyGenostics, Inc., Beijing, China). Genes related to KS included (but were not limited to) ANOS1, NSMF, FGFR1, FGF8, FGF17, IL17RD, PROK2, PROKR2, HS6ST1, CHD7, WDR11, SEMA3A, TUBB3, and SOX10 (Stamou and Georgopoulos, 2018). Genes related to ichthyosis were also included, including (but not limited to) STS, FLG, ABCA12, ALOXE3, ALOX12B, CERS3, CYP4F22, PNPLA1, TGM1, KRT1, KRT10, and KRT2 (Takeichi and Akiyama, 2016). Sequencing was performed using the Illumina HiSeq2000 platform (110-bp paired-end sequencing) according to the standard protocol. The overall sequencing coverage of the target regions was ≥98.95% for a 100× depth of coverage in each chromosome. The coverage of target regions was normalized and compared with the average normalized data of all other samples of the same run to obtain the ratio of the relative coverage in order to detect deletions and duplications in the patients’ genome sequences. The regions where the ratio was below 0.7 were considered putative deletions, while the regions where the ratio rose above 1.3 were speculated to be putative duplications. Variant filtering of the data was done assuming autosomal recessive inheritance according to the pedigree of the consanguineous family. We included frameshift, nonsense, missense, and acceptor and donor splice site variants, as well as variants with minor allele frequency (MAF) of 0.1% in the Single Nucleotide Polymorphism Database (dbSNP build 137), the 1000 Genomes Project, the National Heart, Lung, and Blood Institute (NHLBI), the Exome Sequencing Project Exome Variant Server (EVS), the UCSC common SNP database, and an internal control database using SAMtools (version 1.4) and SOAPsnp software (version 2.0) for further analysis. Whole exome data were deposited and released by the National Center for Biotechnology information (NCBI) database (SRR11745079 for patient 1 and SRR11745080 for patient 2).

## Results

### Clinical Observations

**FIGURE 2:**
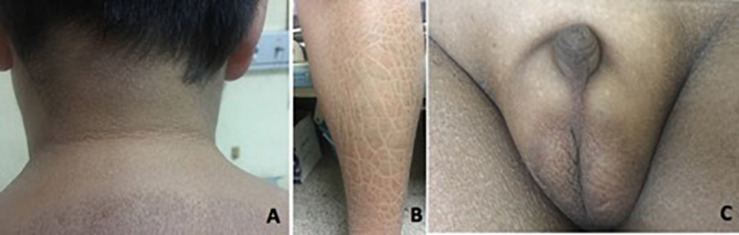
Clinical features in patient 1. **(A)** Acanthosis nigricans can present in the neck. **(B)** Desquamation and gray-brown scaly scales could be seen in the lower extremities. **(C)** Testicular volume was 1 ml bilaterally, with pubic hair P1 and testis length of 1.5 cm.

Patient 1 was referred to our hospital when he was 14 years old, with complaints of absent pubertal development and anosmia. He had bilateral cryptorchidism and had undergone a bilateral orchiopexy at 14 years of age. Physical examination revealed a height of 168 cm (+0 SD), weight of 73 kg (+1.5 SD), body mass index (BMI) of 25.9 kg/m^2^, and waist circumference of 90 cm. His skin was dry. Acanthosis nigricans was observed in the neck and the armpits (Figure 2A). Desquamation and gray-brown scaly skin could be seen in the abdomen and lower extremities (Figure 2B). His breasts were staged as Tanner B3 and pubic hair as P1. His flaccid penile length was 1.5 cm (Figure 2C). Both of his testicular volumes were 1 ml. Ophthalmic examination revealed strabismus, nystagmus, and amblyopia in both eyes. His eyes could follow movement, and convergence was normal. He had undergone surgery for right eye ptosis at 7 years of age. His father is 160 cm tall, and his mother is 155 cm tall.

**FIGURE 3:**
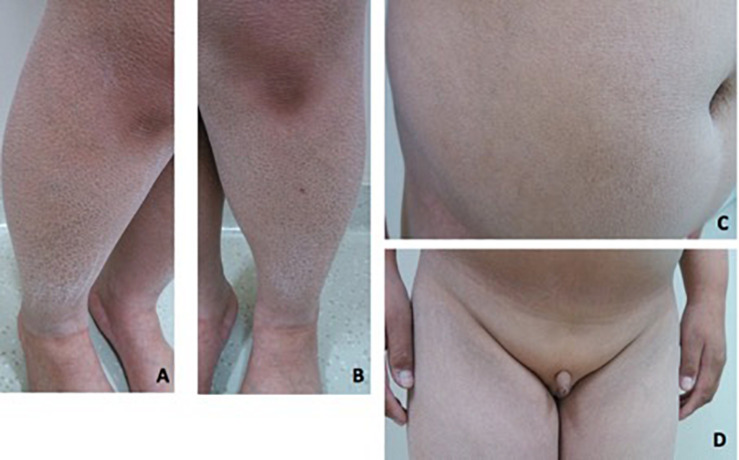
Clinical features in patient 2. **(A–C)** Rough and fish-scaly skin could be seen in the lower extremities and in the abdomen. Patient was abdominal obese. **(D)** Patient had no pubertal development. The penis was 3 cm in the length. The testicle was not palpable.

Patient 2 is a 19-year-old boy. He was referred to our hospital at 14 years of age due to absent pubertal development and a poor sense of smell. On physical examination, he was obese with a height of 153 cm (−1.7 SD), weight of 62 kg (−0.6 SD), and BMI of 26.5 kg/m^2^. Acanthosis nigricans was detected in his neck. Rough and scaly skin could be seen in the neck, abdomen, and lower extremities (Figures 3A–C). His breasts were staged as Tanner B1 and pubic hair as P1. His flaccid penile length was 3 cm. The testicles were not palpable (Figure 3D). Ophthalmic examination indicated strabismus in the left eye and ptosis in the right eyelid. He was 168 cm tall when at 20 years old. His father is 165 cm tall, and his mother is 154 cm tall. His mother had menarche at 13 years old. Both his parents had a normal sense of smell and mental development.

Both patients showed normal mental development. Both patients and their parents denied any behavioral abnormality, abnormal speech development, or autistic behavior. Both patients denied neurological symptoms including headache, dizziness, nausea, vomiting, lack of consciousness, seizure, involuntary movement, or visual defect.

### Diagnostic Laboratory Assessments

In patient 1, the baseline levels of luteinizing hormone (LH) and follicle-stimulating hormone (FSH) were 1.27 IU/L (1.24–8.62) and 1.25 IU/L (1.27–19.26), respectively. LH and FSH increased to 1.83 and 3.50 IU/L, respectively, after administration of a triptorelin stimulating test (100 μg of Triptorelin was intramuscularly injected). Serum lipid profiling indicated a triglyceride (TG) level of 2.29 mmol/L (0.45–1.81), a total cholesterol (TC) level of 3.92 mmol/L (2.9–5.7), and a low-density lipoprotein cholesterol (LDL-C) level of 2.50 mmol/L (2.07–3.12). The results of additional laboratory assessments are shown in Supplementary Table S1.

In patient 2, the baseline levels of LH and FSH were 0.0 IU/L (1.5–9.3) and 0.0 IU/L (1.4–18.1), respectively. LH and FSH increased to 0.24 and 2.18 IU/L, respectively, after administration of a triptorelin-stimulating test (100 μg of Triptorelin was injected intramuscularly). Serum lipid profiling showed a TG level of 0.98 mmol/L (0.45–1.70), a TC level of 2.67 mmol/L (2.85–5.70), and a LDL-C level of 1.58 mmol/L (2.07–3.12). The results of additional laboratory assessments are shown in Supplementary Table S1.

An abdominal ultrasound revealed normal bilateral kidneys in both patients.

### Radiographic Imaging Results

**FIGURE 4:**
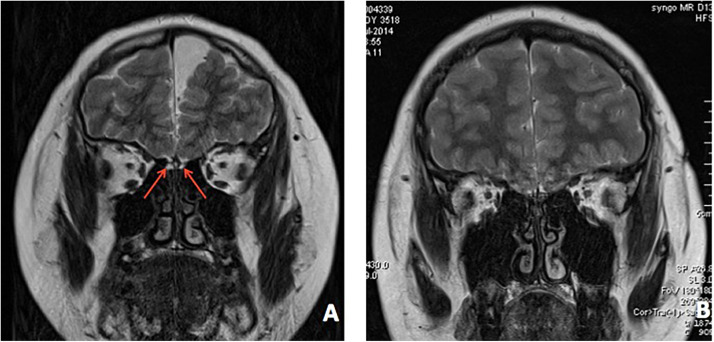
Brain MRI of both patients. **(A)** MRI revealed bilateral dysplasia of olfactory bulb, tract, and sulcu in patient 1. **(B)** MRI revealed bilateral dysplasia of olfactory bulb, tract, and sulcu in patient 2.

In patient 1, an ultrasound found bilateral testis located in the scrotum. An MRI revealed dysplasia of the bilateral olfactory bulb and tract (Figure 4A). The bone age was determined to be 13.5 years old.

In patient 2, an MRI indicated dysplasia of the bilateral olfactory bulb and tract (Figure 4B). His bone age was determined to be 13.5 years old.

### Treatment

An otolaryngology consultation implied that patient 1 had complete anosmia. A dermatology consultation confirmed the diagnosis of ichthyosis. Human chorionic gonadotropin (HCG, 3,000 U) was intramuscularly injected twice per week for 2 weeks, and patient 1’s testosterone level rose from 0.50 to 0.65 ng/ml, indicating a poor response to HCG therapy. A treatment of 80 mg of oral testosterone undecanoate taken three times a day was then administered. One year after beginning oral testosterone treatment, his penis increased to 6 cm in flaccid length. To treat the ichthyosis, a silicone cream was applied externally twice a day and was effective in relieving skin dryness.

For patient 2, oral testosterone undecanoate was given at 40 mg twice a day for 3 months. Then, a combined therapy consisting of HCG 5,000 U and HMG 150 U was intramuscularly injected once a week. One year after beginning treatment, testicular volume had increased to 6 ml bilaterally, and pubic hair had increased to P3 according to the Tanner staging system.

### Pathogenic Mutations

**FIGURE 5:**
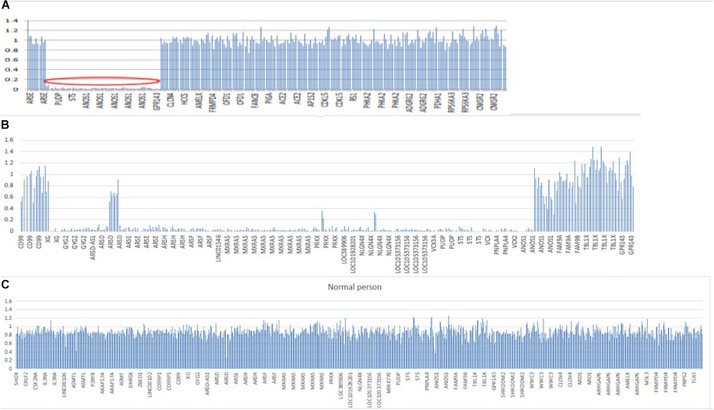
Segmental deletion in the X chromosome in both patients. **(A)** A 3,923-kB deletion within the Xp22.31 regions (chrX: 5810838–9733877) containing GPR143, NLGN4X, VCX, PUDP, PNPLA4, STS, and ANOS1 in patient 1. **(B)** A 5,807-kB deletion within the Xp22.31-Xp22.33 regions (chrX: 2700083–8507807) in patient 2, including the following genes: XG, GYG2, ARSDAS1, ARSD, ARSE, ARSH, ARSF, LINC01546, MXRA5, PRKX, PRKX-AS1, LOC389906, LOC101928201, NLGN4X, LOC105373156, MIR4770, VCX3A, PUDP, MIR4767, STS, VCX, PNPLA4, MIR651, VCX2, VCX3B, and ANOS1. **(C)** Normal people covering the same region on X chromosome. *Y*-axis refers to the ratio of relative coverage of target regions of our patients to that of the average normalized data of all other samples of the same run. The regions where the ratio was below 0.7 were considered putative deletions.

In patient 1, whole exome sequencing analysis identified a 3,923-kb deletion within the Xp22.31 region (chrX:5810838-9733877) compared to normal people (Figures 5A,C). The deleted region contains the *GPR143*, *NLGN4X*, *VCX-A*, *PUDP*, *PNPLA4*, *STS*, and *ANOS1* genes. A heterozygotic missense variant in *NSMF* (c.410A > C, p.Q137P) was also detected. This gene is located at chr9: 140342022–140353786, and mutation of this gene could cause KS inherited in an autosomal dominant mode. The variant was predicted to be likely benign with ACMG classification^1^, damaging with SIFT, probably damaging with Polyphen2 and disease causing with Mutation taster. It was not found in gnomAD, and no publications were found for this variant on VarSome. This gene variant comes from the father (Supplementary Figure S1), who experienced a normal pubertal development. His mother did not demonstrate any pubertal development abnormalities, and both of his parents showed no chromosomal microdeletion.

In patient 2, whole exome sequencing analysis identified a 5,807-kb deletion within the Xp22.31-p22.33 region (chrX: 2700083–8507807) (Figure 5B). The deletion contained 26 genes, including *XG*, *GYG2*, *ARSD-AS1*, *ARSD*, *ARSE*, *ARSH*, *ARSF*, *LINC01546*, *MXRA5*, *PRKX*, *RKX-AS1*, *LOC389906*, *LOC101928201*, *NLGN4X*, *LOC105373156*, *MIR4770*, *VCX3A*, *PUDP*, *MIR4767*, *STS*, *VCX-A*, *PNPLA4*, *MIR651*, *VCX2*, *VCX3B*, and *ANOS1*. His parents did not undergo a whole exome sequencing analysis.

Both of the chromosome deletions on Xp22.31 detected by whole exome sequencing were not listed in the chromosome polymorphism database, the dbVar database, or the Database of Chromosomal Imbalance and Phenotype in Humans using Ensembl Resources (DECIPHER^2^).

### Literature Review

We did a literature review on case reports of KS with XLI, and the genotype–phenotype association of 20 cases was analyzed (Supplementary Table S2). This analysis revealed that 7 of the 20 patients had an intellectual disability (Klink et al., 1994; Martul et al., 1995; Weissörtel et al., 1998; Macarov et al., 2007; Melichar et al., 2007; Cho et al., 2012; Khelifa et al., 2013). Three of these patients had an *NLGN4X* deletion, while three patients did not (Martul et al., 1995; Weissörtel et al., 1998; Macarov et al., 2007; Melichar et al., 2007; Cho et al., 2012; Khelifa et al., 2013), and one case was unclear (Klink et al., 1994). There were three other cases with an *NLGN4X* deletion that demonstrated normal mental development (Mochel et al., 2008; Liu et al., 2016; Nagai et al., 2017). Previous literature indicated that the relationship between *NLGN4X* mutations and intellectual disability could not be definitively established (Khelifa et al., 2013). A previous summary pointed out that, among the nine patients with *XLI* reported in the literature (Khelifa et al., 2013), five patients had different degrees of intellectual disability, only one of which had a deletion of *NLGN4X.* Another patient who had a deletion of *NLGN4X* demonstrated normal mental development (Khelifa et al., 2013). Renal agenesis occurred in 8 of the 20 patients, which may be caused by an *ANOS1* deletion (Martul et al., 1995; Krishnamurthy et al., 2007; Macarov et al., 2007; Trevisson et al., 2015; Xu et al., 2015; Nagai et al., 2017). Previous literature also noted that patients with KS harboring a deletion in Xp22.3 were more likely to exhibit renal agenesis (Kirk et al., 1994). Among 17 patients with either X-linked KS or X-linked KS and XLI, 6 patients had only one kidney (Kirk et al., 1994). Obesity occurred in 5 of the 20 patients (Martul et al., 1995; Weissörtel et al., 1998; Cho et al., 2012; Khelifa et al., 2013; Berges-Raso et al., 2017). Among four cases with deletion of *PNPLA4*, two patients were obese, and one had fatty liver and hyperlipidemia (Cho et al., 2012; Khelifa et al., 2013; Liu et al., 2016; Nagai et al., 2017). Among the 11 cases with short stature, two had deletions of SHOX while three had deletions of *ARSE* (Meindl et al., 1993; Martul et al., 1995; Maya-Núñez et al., 1998; Weissörtel et al., 1998; Krishnamurthy et al., 2007; Melichar et al., 2007; Mochel et al., 2008; Cho et al., 2012; Liu et al., 2016; Nagai et al., 2017). Three of the 20 patients presented with chondrodysplasia punctate, of whom 2 cases had deletions in *ARSE* and 1 case was unclear (Meindl et al., 1993; Melichar et al., 2007; Cho et al., 2012). Among the three cases with X-linked ocular albinism, two cases had a deletion of *GPR143* (Melichar et al., 2007; Cho et al., 2012), and one case was unclear. One out of the 20 cases showed strabismus, but only deletion of *STS* and *ANOS1* was detected (Martul et al., 1995).

## Discussion

Our study recruited two KS patients presenting with XLI, obesity, and strabismus. A novel 3,923 kb deletion within the Xp22.31 region containing 7 genes and a novel 5,807 kb deletion within the Xp22.31–Xp22.33 region containing 26 genes were identified. Cytogenetically visible deletions of the X chromosome from Xp22.2 to Xpter may lead to nullisomy of the deleted region and result in variable contiguous gene syndromes. To date, several apparently unrelated phenotypes have been found to associate with certain gene deletions within Xp22.3 regions, including *SHOX* in regard to short stature, *ARSE* in regard to chondrodysplasia punctata, *STS* in regard to XLI, *ANOS1* in regard to KS, *GPR143* in regard to ocular albinism type 1 (OA1), *VCX-A* in regard to intellectual disability, and *NLGN4X* in regard to intellectual disability in conjunction with autism (Krishnamurthy et al., 2007; Macarov et al., 2007; Mochel et al., 2008; Cho et al., 2012; Ben Khelifa et al., 2013; Trevisson et al., 2015; Xu et al., 2015; Liu et al., 2016; Nagai et al., 2017).

Both patients had phenotypes consistent with KS and XLI, which may be explained by the deletion of *ANOS1* and *STS*. *ANOS1* is the first gene found to be associated with KS and initially identified in X-linked KS (de Castro et al., 2014, 2017). Approximately 5–7% of patients with KS had a causative *ANOS1* mutation. Mutations in *ANOS1* are typically either nonsense mutations, frame shift mutations, or large gene deletions (Senthilraja et al., 2019). *ANOS1* contains 14 exons and encodes the extracellular adhesion protein amosmin-1, which mediates adhesion and axonal migration of GnRH neurons (Soussi-Yanicostas et al., 2002; Bribián et al., 2006; Gianola et al., 2009; Yanicostas et al., 2009; de Castro et al., 2014, 2017; García-González et al., 2016; Murcia-Belmonte et al., 2016; Stamou and Georgopoulos, 2018). Anosmin-1 is largely present in different structures of the central nervous system, including the cerebral cortex, olfactory bulb, and other components of the olfactory system, and when mutated in KS may lead to satellite symptoms including anosmia and HH (García-González et al., 2016; de Castro et al., 2017; Stamou and Georgopoulos, 2018). Anosmin-1 was shown to play essential roles in the patterning of mitral and tufted cell axon collaterals to the olfactory cortex (Soussi-Yanicostas et al., 2002) and also interacts with FGFR1 (alias, KAL-2), thereby impairing the migration of oligodendrocyte precursors and producing substantial morphological changes in the new subventricular zone (Bribián et al., 2006; García-González et al., 2010; Esteban et al., 2013; Murcia-Belmonte et al., 2014; García-González et al., 2016). New hypotheses have been described to explain both the main and satellite neurological symptoms of KS with regard to alterations in myelination caused by anosmin-1 (Garcia-Gonzalez et al., 2013). Although different putative interacting proteins and mechanisms have been deeply studied (FGFR1, heparan sulfates, syndecans, glypicans, uPA, fibronectin, laminin, integrin-beta), the current understanding of the mechanism of anosmin-1 in KS remains far from complete (Soussi-Yanicostas et al., 2002; Dodé et al., 2003; Bribián et al., 2006; Gianola et al., 2009; Yanicostas et al., 2009; García-González et al., 2010, 2016; Esteban et al., 2013; Garcia-Gonzalez et al., 2013; Murcia-Belmonte et al., 2014; Murcia-Belmonte et al., 2016). The complete deletion of *ANOS1* is rare (Hamada et al., 2013). Compared with patients with a point mutation in *ANOS1*, patients with complete deletion of *ANOS1* may present with other symptoms, such as short stature, chondrodysplasia punctata, intellectual disability, and steroid sulfatase deficiency (Hamada et al., 2013). These symptoms may be caused by deletions in genes located close to *ANOS1*. *STS* is located near *ANOS1* and encodes a steroid sulfatase enzyme that catalyzes the conversion of steroid sulfate precursors to estrogens, and defects in this process can cause XLI (Hamada et al., 2013).

The deleted fragments detected in patients 1 and 2 had six genes in common, including *NLGN4X*, *VCX-A*, *PUDP*, and *PNPLA4*, in addition to *STS* and *ANOS1*. *GPR143* (*OA1* gene), only identified as deleted in patient 1, has been linked to X-linked ocular albinism (OA1; OMIM 300500) (9). Mutations in *GPR143* cause Nystagmus 6, which presents with conjugate, horizontal oscillations of the eyes. Other associated features may include mildly decreased visual acuity, strabismus, and astigmatism. We suspected the association of *GPR143* with strabismus in patient 1, but a family proband with strabismus revealed a 7.7-Mb deletion within Xp22.2–Xp22.3 that did not encompass *GPR143.* Mutation of *NLGN4X* is associated with intellectual disability (OMIM: 300495), X-linked Asperger syndrome type 2 (OMIM: 300497), and autism (Daoud et al., 2009; Shi et al., 2013). *VCX-A* and *VCX-3A* deficiency has been previously shown to be associated with intellectual disability (Chocholska et al., 2006). Although both patients had *NLGN4X* and *VCX-A* deletions, neither had speech defects, intellectual disability, autism, or social disorders. Previous observations also indicated that the deletion of *NLGN4X* may be associated with normal mental development (Khelifa et al., 2013; Nagai et al., 2017). *VCX*-A deletion is associated with XLI (Van Esch et al., 2005; Cuevas-Covarrubias and González-Huerta, 2008), poor sperm production, and sexual development abnormalities (Zou et al., 2003). *PNPLA4* encodes a patatin-like phospholipase with triacylglycerolase and transacylase activity, which may be related to maintaining the homeostasis of adipocyte triglycerides. *PNPLA4* deletion has been reported in patients with XLI (Murugesan et al., 2013). Therefore, the remarkable elevation of triglyceride levels in patient 1 and obesity in both patients may be associated with loss of *PNPLA4*.

In patient 2, a 5,807-kb deletion was identified including *XG*, *GYG2*, *ARSD-AS1*, *ARSD*, *ARSE*, *ARSH*, *ARSF*, *LINC01546*, *MXRA5*, *PRKX*, *PRKX-AS1*, *LOC389906*, *LOC101928201*, *NLGN4X*, *LOC105373156*, *MIR4770*, *VCX3A*, *PUDP*, *MIR4767*, *STS*, *VCX-A*, *PNPLA4*, *MIR651*, *VCX2*, *VCX3B*, and *ANOS1*. In 1986, Ballabio et al. (1986) reported that variations in the *XG* gene were associated with XLI. The *GYG2* gene has a homologous sequence located on the Y chromosome, indicating that there may be compensation for the function of this gene (Zhai et al., 2000). *ARSD*, *ARSE*, and *ARSF* are members of the sulfatase family, which help maintain a crucial component of the bone and cartilage matrix. Studies have confirmed that X-linked punctate cartilage dysplasia is associated with *ARSE* and *ARSF* mutations (Franco et al., 1995; Brunetti-Pierri et al., 2003). Loss of these genes may be related to short stature. *ARSE* is responsible for chondrodysplasia punctata, which presents with punctate cartilage dysplasia, dwarfism, maxillary hypoplasia, hearing impairment, point calcification of the epiphysis, and cataracts. *ARSH* is involved in the synthesis of hormones, regulation of signaling pathways, and macromolecular degradation (Sardiello et al., 2005). Patient 2 presented short stature at initial diagnosis but achieved an adult height (AH) consistent with his parents’ height at later follow-up appointments and did not show any punctate cartilage dysplasia. Both patients in this study achieved a greater AH than their parental target height, in accordance with previous findings that KS patients tend to have greater AH compared to the general population, independent of specific genetic causes (Maione et al., 2020). *MXRA5* encodes proteins associated with extracellular matrix remodeling (Balakrishnan et al., 2014). *VCX3A*, only expressed in male germ cells, may mediate sperm production, whose defect is associated with XLI and may be associated with intellectual disability (Van Esch et al., 2005; Cuevas-Covarrubias and González-Huerta, 2008; Khelifa et al., 2013). A summary was generated to indicate the gene function, phenotype, and related diseases (Supplementary Table S3).

Mutation in *NSMF* may cause KS in an autosomal dominant inheritance mode. However, we discovered a likely benign heterogeneous missense variant of *NSMF* (c.410A > C, p.Q137P) in patient 1, which was derived from the father, who had experienced normal pubertal development. Therefore, we supposed that this variation did not contribute to the pathogenesis of KS in patient 1 (Stamou and Georgopoulos, 2018).

Both patients revealed bilateral dysplasia of the olfactory bulb, tract, and sulcus by MRI, which are the most common findings in KS patients. MRI findings have been shown to have great accuracy and strong consistency when combined with smell test in differentiating KS patients from nIHH patients (Koenigkam-Santos et al., 2010; Koenigkam-Santos et al., 2011). A recent study that further analyzed MRI differences found that patients with KS showed differences in white matter between patients with and without mirror movements (Koenigkam-Santos et al., 2010).

The use of comparative genomic hybridization (CGH) arrays has increased the identification of genomic rearrangements and is an essential tool for the detection of microchromosomal deletions. Whole exome sequencing is the most frequently used genome sequencing method used to identify mutations in coding regions and untranslated regions (UTRs) related to disease and population evolution. We chose to use whole exome sequencing in this study because previous literature has indicated the possibility of a genomic rearrangement involving the *ANOS1* gene and a point mutation of the *STS* gene leading to KS and XLI (Trevisson et al., 2015).

Both of our patients are male patients. The prevalence of KS in male individuals greatly outweighs that in female individuals. This may be because *ANOS1* partially escapes X inactivation (Dodé et al., 2003). Female patients may therefore carry the deletion but not show an accordant phenotype (Chocholska et al., 2006). The onset age of both our patients is 14 years old, which is consistent with the majority of previous case reports (Krishnamurthy et al., 2007; Macarov et al., 2007; Mochel et al., 2008; Cho et al., 2012; Khelifa et al., 2013; Trevisson et al., 2015; Xu et al., 2015; Liu et al., 2016; Nagai et al., 2017). KS often presents with delayed pubertal development, and patients tend to be identified by clinicians during the period of puberty.

## Conclusion

We identified two patients presenting with KS, ichthyosis, obesity, and strabismus caused by novel microdeletions in the X chromosome. Loss of *ANOS1* and *STS* is known to cause KS and ichthyosis. Obesity may be associated with loss of the *PNPLA4* gene. Our research shows a novel microdeletion in the X chromosome that leads to Kallmann–ichthyosis phenotype and also discovered that the observed phenotypes of obesity and strabismus may be caused by other gene deletions in the region, which requires further studies to better understand the relationship of these deletions to KS and XLI. For clinical endocrinologists, symptoms of obesity, strabismus, and ichthyosis should be evaluated in patients with X-linked recessive KS, which may be caused by genes located close to *ANOS1*.

## Data Availability Statement

We have uploaded data to NCBI SRA data base with accession numbers SRR11745079 and SRR11745080.

## Ethics Statement

The studies involving human participants were reviewed and approved by the Ethics Committee of PUMCH. Written informed consent to participate in this study was provided by the participants’ legal guardian/next of kin. Written informed consent was obtained from the individual(s), and minor(s)’ legal guardian/next of kin, for the publication of any potentially identifiable images or data included in this article.

## Author Contributions

WM designed and carried out the study and wrote the manuscript. JM, XWu, MN, and XWa designed the study and revised the manuscript. JM, XWu, LD, YS, and XL provided patient and sample. JZ helped carry on the study and contact patient. ZL contacted patient. All authors contributed to the article and approved the submitted version.

## Conflict of Interest

The authors declare that the research was conducted in the absence of any commercial or financial relationships that could be construed as a potential conflict of interest.

## Footnotes

1 https://varsome.com/

2 http://www.sanger.ac.uk/PostGenomics/decipher/

## Supplementary Material

FIGURE S1 Variation NSMF gene, inherited from his father. Figure **(A)** shows mutation in NSMF (c.410A > C and p.Q137P) chr9: 140342022-140353786 in patient I. The mutation would cause Kallmann syndrome in an autosomal dominant heritage model. It was also detected in his father shown by **(B)** who experienced normal pubertal development. **(C)** shows that the mother doesn’t have the mutation.

TABLE S1 Laboratory assessments of patient 1 and 2 β-HCG: β-chorionic gonadotropin; 17a-OHP:17a hydroxyl-progesterone; DHEAS: Dehydroepiandrosterone Sulfate; 24hUFC:24h urine free cortisol; IGF-1: insulin-like growth factors-1; FT3: Free Triiodogonine; FT4: Free Thyroxine; TSH: Thyrotropin; F: Serum-free cortisol; ACTH: Adrenocorticotropic hormone; FSH(60min) and LH(60min): gonadotropins levels after triptorelin stimulating test.

TABLE S2 Literature review of 10 cases of KSand XLI.

TABLE S3 The function, relevant disease and phenotype ofgenes on Xp 22.3. KS:Kallmann Syndrome; XLI:X-linked ichthyosis.
