# Supplementary figures and images for: Novel Microdeletion in the X Chromosome Leads to Kallmann Syndrome, Ichthyosis, Obesity, and Strabismus
Source: Front Genet. 2020 Jun 24;11:596. doi: 10.3389/fgene.2020.00596 (PMC7327112; doi:10.3389/fgene.2020.00596)

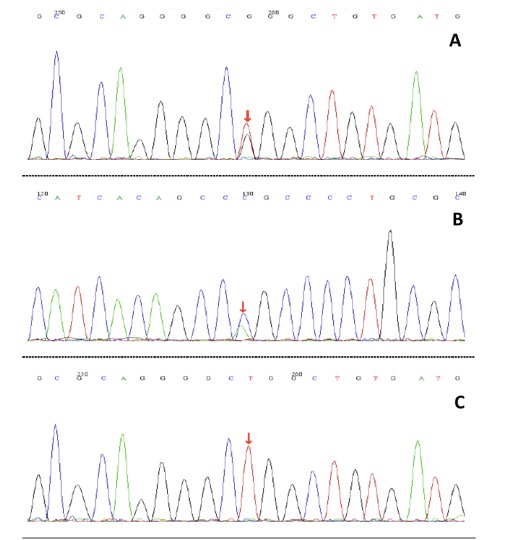

Supplement: FIGURE S1 — Variation NSMF gene, inherited from his father. Figure (A) shows mutation in NSMF (c.410A > C and p.Q137P) chr9: 140342022-140353786 in patient I. The mutation would cause Kallmann syndrome in an autosomal dominant heritage model. It was also detected in his father shown by (B) who experienced normal pubertal development. (C) shows that the mother doesn’t have the mutation. [file Figure_1.jpg]
