# Supplementary material for: Novel Microdeletion in the X Chromosome Leads to Kallmann Syndrome, Ichthyosis, Obesity, and Strabismus
Source: Front Genet. 2020 Jun 24;11:596. doi: 10.3389/fgene.2020.00596 (PMC7327112; doi:10.3389/fgene.2020.00596)
Supplement: TABLE S1 — Laboratory assessments of patient 1 and 2 β-HCG: β-chorionic gonadotropin; 17a-OHP:17a hydroxyl-progesterone; DHEAS: Dehydroepiandrosterone Sulfate; 24hUFC:24h urine free cortisol; IGF-1: insulin-like growth factors-1; FT3: Free Triiodogonine; FT4: Free Thyroxine; TSH: Thyrotropin; F: Serum-free cortisol; ACTH: Adrenocorticotropic hormone; FSH(60min) and LH(60min): gonadotropins levels after triptorelin stimulating test. [file Table_1.docx]

| Hormones | Patient 1 | Patient 2 | Reference range |
| --- | --- | --- | --- |
| LH(0min)(IU/L) | 1.3 | 0.0 | 1.2-8.6 |
| FSH(0min)(IU/L) | 1.6 | 0.0 | 1.3-19.3 |
| LH(60min)(IU/L) | 1.8 | 0.2 | 12.6-48.8 |
| FSH(60min)(IU/L) | 3.5 | 2.2 | 11.8-24.3 |
| Estradiol (pg/ml) | 36 | 20 | ＜47 |
| Progesterone (ng/ml) | 1.42 | 0.22 | 0.10-0.84 |
| Testosterone (ng/ml) | 0.57 | 0.06 | 1.75-7.81 |
| Prolactin (ng/ml) | 5.7 | 2.0 | 2.6-13.1 |
| β-HCG (IU/l) | 0.2 | 0.0 | ＜5.0 |
| FT4 (ng/dl) | 1.09 | 1.41 | 0.81-1.89 |
| FT3 (pg/ml) | 2.58 | 2.81 | 1.80-4.10 |
| TSH(uIU/ml) | 5.85 | 2.06 | 0.38-4.34 |
| IGF1 (ng/ml) | 175 | 154 | 101-267 |
| Growth hormone (ng/ml) | 0.4 | 1.1 | < 2.0 |
| F (8am) (ug/dl) | 12.7 | 5.8 | 4.1-22.3 |
| ACTH(8am) (pg/ml) | 105 | 29 | 0-46 |
| 24hUFC (μg) | 39.6 | - | 12.3-103.5 |
| DHEAS(ug/dl) | 132 | - | ＜312 |
| 17a-OHP(ng/ml) | 0.28 | 0.11 | 0.31-2.17 |
| Fasting insulin (uIU/ml) | 6.9 | 11.0 | 5.2-17.2 |

Supplementary Table1. Laboratory assessments of patient 1 and 2

β-HCG: β-chorionic gonadotropin; 17a-OHP: 17a hydroxyl-progesterone; DHEAS: Dehydroepiandrosterone Sulfate; 24hUFC: 24h urine free cortisol; IGF-1: insulin-like growth factors-1; FT3: Free Triiodogonine; FT4: Free Thyroxine; TSH: Thyrotropin;F: Serum-free cortisol; ACTH: Adrenocorticotropic hormone; FSH(60min) and LH(60min): gonadotropins levels after triptorelin stimulating test;
