# Supplementary material for: Novel Microdeletion in the X Chromosome Leads to Kallmann Syndrome, Ichthyosis, Obesity, and Strabismus
Source: Front Genet. 2020 Jun 24;11:596. doi: 10.3389/fgene.2020.00596 (PMC7327112; doi:10.3389/fgene.2020.00596)
Supplement: TABLE S2 — Literature review of 10 cases of KSand XLI. [file Table_2.docx]

| Cases | Sex/Age | Deleted  Region | Deleted  Length | Deletion gene | Chondrodysplasia punctata | Ocular albinism | Renal  agenesis | Obesity | Short  Stature | Strabismus | Intellectual disability |
| --- | --- | --- | --- | --- | --- | --- | --- | --- | --- | --- | --- |
| Berges-Raso  et al.,2017 | M/32y | Xp22.3 | / | / | - | - | / | + | / | - | - |
| Nagai  et al.,2017 | M/6-months | Xp22.31 | 2.7Mb | NLGN4X, VCX3A,HDHD1, STS, VCX, PNPLA4, VCX2, VCX3B, exons 8–14 of ANOS1 | - | - | +a | - | + | - | - |
| Liu  et al.,2016 | M/24y | Xp22.31 | 5.4 Mb | XG、GyG2、ARSD、ARSE、ARSH、ARSF、MXRA5、PPKX、NLGN4X、VCX3A、  HDHD1A、STS、VCX、PNPLA4 | - | - | / | / | + | - | - |
| Trevisson  et al.,2015 | M/9y | xp22.31 | 518 kb | ANOS1, hemizygous missense variant c.1393A > G p.(Lys465Glu) in STS exon 10 | - | - | +b | / | / | - | - |
| Xu et al. ,2015 | M/  Unknown | Xp22.3 | 1596 kb | exons 9-14 of ANOS1 and entire STS (6938911–8 535148) | - | - | +c | / | - | - | - |
| Ben Khelifa et al.,2013 | M/14y | Xp22.31 | 2Mb | VCX3A,HDHD1A,STS,VCX,PNPL4,VCX2(6395312-8383288) | - | - | / | + | - | - | + |
| Cho et al.,2012 | M/13.5y | Xp22.2 | 9.7Mb | PPP2R3B,SHOX,CRLF2,CSF2RA,IL3RA,SLC25A6,P2RY8,AKAP17A,ASMT,DHRSX,  ZBED1,CD99P1,CD99,XG,GYG2,ARSD,ARSE,ARSH,ARSF,PRKX,NLGN4X,VCX3A,  HDHD1A,STS,VCX,PNPLA4,VCX3B,ANOS1,TBL1X,GPR143(telomere of Xp to GPR143 of Xp22) | + | + | / | +d | + | - | + |
| Mochel et al.,2008 | M/17y | Xp22.3 | 3.7Mb | NLGN4X, VCX-3A, VCX-1, VCX-2, VCX-3B, STS and ANOS1 | - | - | - | / | + | - | - |
| Krishnamurthy et al.,2007 | M/10y | Xp22.3 | / | STS | - | - | +e | / | + | - | / |
| Macarov et al., 2007 | M/2.4y | Xp22.3 | 4.5Mb | ARSE,DXS31,DXS89,DXS1060,NLGN4,DXS6837,VCX-A,DXS7742,GS1,STS,VCX-B1,GS2,VCX-B,VCX-C,ANOS1,DXS1467,DXS8051 | - | - | +f | / | / | - | + |
| Melichar et al.,2007 | M/38  Weeks | Xp22.31 | 9.6Mb | SHOX, ARSE, NLGN4, STS, ANOS1, and GPR143 | +g | + | - | - | + | - | + |
| Weissörtel et al.,1998 | M/15.4y | Xp22.3 | 3.5Mb | DXS996,DXSl118E, BW 14, KAL (Exon 2) and KAL(Exon 13) | - | - | - | + | + | - | + |
| Maya-Nuñez et al.,1999 | M/30y | Xp22.3 | / | STS | / | / | / | / | - | / | / |
| Maya-Nuñez et al.,1999 | M/10y | Xp22.3 | / | STS | / | / | / | / | - | / | / |
| Maya-Nuñez et al.,1998 | M/20y | Xp22.3 | / | STS, exons 1-3 of ANOS1 | - | - | - | - | + | - | - |
| Martul et al.,1995 | M/14y | Xp22.3 | 2Mb | STS,ANOS1 | - | - | + | - | - | - | + |
| Martul et al.,1995 | M/13y | Xp22.3 | 2Mb | STS,ANOS1 | - | - | + | - | + | - | - |
| Martul et al.,1995 | M/41y | Xp22.3 | 2Mb | STS,ANOS1 | - | - | + | + | + | + | - |
| Klink et al.,1994 | M/  Unknown | Xp22.3 | 3.18-3.57Mb | / | - | - | - | - | - | - | + |
| Meindl et al.,1993 | M/9y | Xp22.3 | 10Mb | / | +h | + | - | - | + | - | - |

Supplementary Table 2. Literature review of 10 cases of KS and XLI

a.Right renal aplasia and left hydronephrosis

b.Left kidney agenesis

c.Right renal agenesis

d. Fatty liver,hyperlipidemia,DM,cleft lip

e.Absent left kidney, nephrotic syndrome

f.Bimanual synkinesa

g. Sensorineural deafness

h: mirror movement

"／" Not mentioned in the paper, "- "not present for the symptom,"+" present for the symptom
