# Supplementary material for: Novel Microdeletion in the X Chromosome Leads to Kallmann Syndrome, Ichthyosis, Obesity, and Strabismus
Source: Front Genet. 2020 Jun 24;11:596. doi: 10.3389/fgene.2020.00596 (PMC7327112; doi:10.3389/fgene.2020.00596)
Supplement: TABLE S3 — The function, relevant disease and phenotype ofgenes on Xp 22.3. KS:Kallmann Syndrome; XLI:X-linked ichthyosis. [file Table_3.docx]

| Genes | Functions | Possibly relevant diseases and phenotypes |
| --- | --- | --- |
| *ANOS1* | Encoding extracellular adhesion protein anosmin-1, which mediates adhesion and axonal migration of GnRH neurons | KS; anosmia; renal dysplasia; mirror movement; cleft lip |
| *STS* | Encoding a steroid sulfatase enzyme that catalyzes the conversion of sulfated steroid precursors to the free steroid | XLI |
| *GPR143* | Encoding a protein involved in intracellular signal transduction mechanisms that binds to heterotrimeric G proteins and is targeted to melanosomes in pigment cells | ocular albinism type 1,Nystagmus 6,decreased visual acuity, strabismus, and astigmatism |
| *NLGN4X* | Shearing of specific sites of G-axon protein, formation and remodeling of central nervous system synapses | Intellectual disability, X-linked Asperger syndrome type 2 and autism |
| *VCX-A/VCX-3A* | Expressed in male germ cells | Intellectual disability, XLI, poor sperm production and sexual development abnormalities |
| *HDHD1(PUDP)* | Member of haloacid dehalogenase-like hydrolase | / |
| *PNPLA4* | Encoding potato-like phospholipase with triacylglycerolase and transacylase activity | Elevation of triglyceride level and obesity |
| *XG* | Encoding Xg antigen | XLI |
| *GYG2* | Encoding glycogenin, involved in glycogen synthesis | / |
| *ARSD,ARSE,ARSF* | Bone and cartilage matrix synthesis | X-linked chondrodysplasia punctata |
| *ARSH* | Arylsulfatase enzyme family, involved in the synthesis of hormones, regulation of signaling pathways and macromolecular degradation | / |
| *MXRA5* | Extracellular matrix remodeling | / |

Supplementary Table 3. The function, relevant disease and phenotype of genes on Xp 22.3

KS: Kallmann Syndrome; XLI:X-linked ichthyosis

"/":Unclear
